# *Plasmodium* sporozoites trickle out of the injection site

**DOI:** 10.1111/j.1462-5822.2006.00861.x

**Published:** 2007-05-01

**Authors:** Lucy Megumi Yamauchi, Alida Coppi, Georges Snounou, Photini Sinnis

**Affiliations:** 1 Department of Medical Parasitology, New York University School of Medicine 341 East 25th Street, New York, NY 10010, USA.; 2 Parasitolgie Comparée et Modèle Expérimentaux, USM 0307, CNRS IFR101, Muséum National d'Histoire Naturelle CP52, 61 Rue Buffon, 75231 Paris Cedex 05, France.

## Abstract

*Plasmodium* sporozoites make a remarkable journey from the skin, where they are deposited by an infected Anopheline mosquito, to the liver, where they invade hepatocytes and develop into exoerythrocytic stages. Although much work has been done to elucidate the molecular mechanisms by which sporozoites invade hepatocytes, little is known about the interactions between host and parasite before the sporozoite enters the blood circulation. It has always been assumed that sporozoites rapidly exit the injection site, making their interactions with the host at this site, brief and difficult to study. Using quantitative PCR, we determined the kinetics with which sporozoites leave the injection site and arrive in the liver and found that the majority of infective sporozoites remain in the skin for hours. We then performed sub-inoculation experiments which confirmed these findings and showed that the pattern of sporozoite exit from the injection site resembles a slow trickle. Last, we found that drainage of approximately 20% of the sporozoite inoculum to the lymphatics is associated with a significant enlargement of the draining lymph node, a response not observed after intravenous inoculation. These findings indicate that there is ample time for host and parasite to interact at the inoculation site and are of relevance to the pre-erythrocytic stage malaria vaccine effort.

## Introduction

Infection of the mammalian host with malaria is initiated when *Plasmodium* sporozoites, present in the salivary glands of an infected *Anopheles* mosquito, are introduced into the skin with the saliva as the mosquito probes for blood. The sporozoites enter the blood circulation and then reach and invade their unique target cell, the hepatocyte. At the end of this obligatory, clinically silent and relatively short hepatic multiplication step, merozoites are released into the blood stream where they invade and multiply in red blood cells. All the clinical symptoms of malaria are associated with the erythrocytic phase of the infection which generally lasts for many months in the absence of treatment. Thus, the pre-erythrocytic stages, when parasite numbers are comparatively low, provide an excellent target for vaccines aimed at preventing infection (Sinnis and Nussenzweig, 1996).

It has long been thought that sporozoites reach the liver quickly following the mosquito bite. Indeed, sub-inoculation experiments in humans have demonstrated that sporozoites appear in the circulation even while the infected mosquito is feeding (Fairley, 1947). In addition, the demonstration, *in vitro*, that sporozoite infectivity wanes rapidly following incubation at 37°C, has suggested that this infective stage must reach its target organ within 1 h (Vanderberg, 1975). Thus, the perception is that sporozoites as well as merozoites, the other infective form of *Plasmodium* in the mammalian host, must quickly invade their target cell, before they lose infectivity or are destroyed by the host immune system.

Recent studies show that the majority of sporozoites are injected into the skin of the mammalian host and not directly into the blood circulation (Sidjanski and Vanderberg, 1997; Matsuoka *et al*., 2002; Medica and Sinnis, 2005), a finding that is not unexpected given that mosquitoes salivate while they are probing for blood but not when they are imbibing blood. After their injection into the dermis, video microscopic observations of fluorescent *Plasmodium berghei* sporozoites elegantly demonstrated that sporozoites move randomly in the dermis until contacting dermal blood vessels which they penetrate, thus entering the blood circulation and exiting the inoculation site (Amino *et al*., 2006). In the present study, we analysed the fate of sporozoites deposited in the skin over an extended period of time using techniques that allowed us to determine how long infectious sporozoites reside in this location.

## Results

**Fig. 1:**
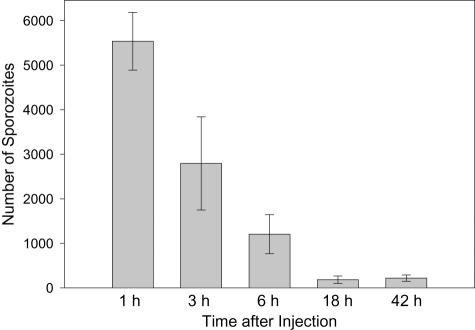
Kinetics with which sporozoites disappear from the inoculation site. Mice were injected intradermally in the ear with 5000 *P. yoelii* sporozoites, the ears were removed at the indicated time points and the number of sporozoites in each ear was quantified by PCR. There were four mice per group and the means ± standard deviations are shown.

In the following experiments we used the rodent parasite *Plasmodium yoelii* because infectivity of its sporozoites to laboratory rodents is significantly higher than that of *P. berghei* (Weiss, 1990; Khan and Vanderberg, 1991; Khusmith *et al*., 1991), thus allowing us to conduct meaningful quantitative studies of hepatic parasite loads. We first wished to establish the kinetics of sporozoite exit out of the inoculation site. Five thousand *P. yoelii* sporozoites dissected from salivary glands were injected intradermally into the distal portion of the pinna of one ear of each mouse. At different time points after injection, the segment of the ear in which the sporozoites were introduced was removed and the number of remaining sporozoites was estimated by quantitative PCR. In preliminary studies, no decrease in the sporozoite loads could be noted when the ears were harvested at 10 and 30 min after injection (data not shown). The observation period was therefore extended. As shown in Fig. 1, most of the injected sporozoites were still present in the ear 1 h after inoculation, and a significant decrease in sporozoite numbers was observed 3 h post injection. The large variation in the number of sporozoites present in the 3 h samples was consistently observed (four experiments with four mice/group/experiment), suggesting that at this time, the majority of sporozoites were in the process of leaving the inoculation site or being destroyed. It is interesting to note that a minor proportion of sporozoites could still be reproducibly detected in the ear at 18 and 42 h post injection.

In the above experiments, it was not possible to distinguish between removal of parasites by *in situ* destruction (i.e. via innate host responses) or by their successful entry into the blood circulation. We employed two complementary approaches to address this question, namely reverse transcription followed by quantitative PCR (RT-PCR) and sub-inoculation.

**Fig. 2:**
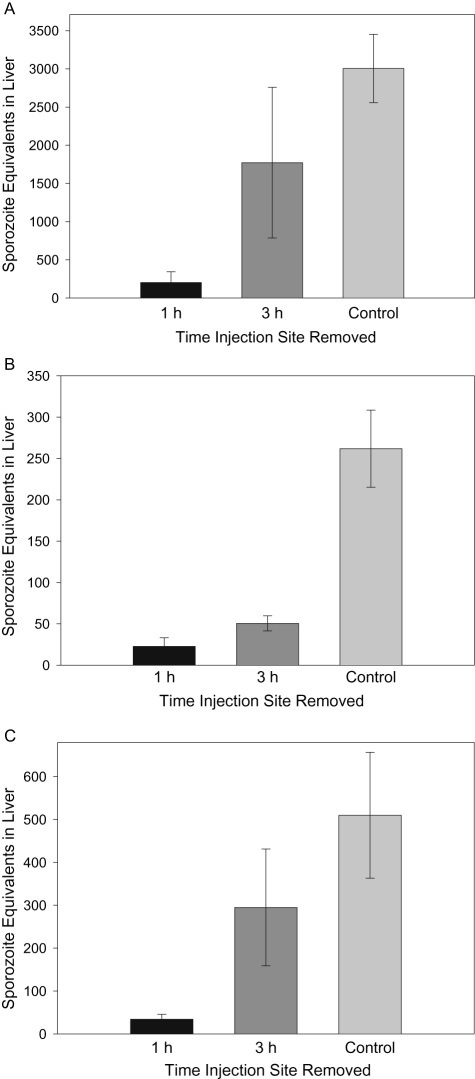
Kinetics of sporozoite arrival in the liver. Mice were inoculated intradermally in the ear with 4000 *P. yoelii* sporozoites (A), exposed to 20 infective mosquito bites, 10 in each ear (B), or 20 infective mosquito bites in the back (C) and the injection site was removed at the indicated time points. Control mice had the uninjected ear removed at 3 h (A), no ears removed (B) or an adjacent uninjected site removed at 3 h (C). Forty hours after sporozoite inoculation, mice were sacrificed and liver parasite burden was quantified by RT-PCR. There were five (A) or eight (B and C) mice per group and the means ± standard deviations are shown.

In the first set of experiments, the intradermal inoculation site was removed at different time points after injection and the parasite liver load was determined 40 h later by RT-PCR. Using this methodology, only sporozoites which have invaded hepatocytes and undergone many cycles of replication are detectable because the small amount of rRNA present in the injected sporozoites or in early liver stages is below the sensitivity of the assay (Briones *et al*., 1996). As shown in Fig. 2A, the majority of inoculated sporozoites that would ultimately productively infect the liver, took over 1 h to exit the dermis. By 3 h post injection most of the infective sporozoites had left the site; however, we consistently observed a large amount of variability in liver stage infection when the injection site was removed at this time point (five experiments, five mice/group/experiment). This variability parallels the variable numbers of sporozoites remaining at the injection site at this time point (Fig. 1).

In order to determine whether this pattern of sporozoite release occurs under natural conditions of transmission, i.e. through mosquito bite rather than by needle injection, we repeated the experiments by allowing 20 infected mosquitoes to feed on each mouse. We selected two different sites for biting that differ in the extent of vascularization: the ear and the back. Overall, the pattern of sporozoite release observed when sporozoites were injected by mosquitoes was similar to what we found with needle injection (Fig. 2A–C). However, when sporozoites were injected by mosquito bite into the ear, fewer parasites had left the site at 3 h post inoculation compared with sporozoites that were injected either by needle into the ear or by mosquito bite into the back. This difference might reflect the lower density of blood vessels in the ear compared with the back and indicates that tissue damage due to needle insertion might have accelerated the rate of sporozoite exit from the ear. Taken together, the data clearly show that regardless of the inoculation site, sporozoites do not leave the injection site in appreciable numbers before 1 h. After this time point, however, the kinetics of sporozoite exit may depend upon factors, such as the density of the vascular bed and the extent of capillary damage, which may influence the ease with which the blood circulation can be accessed. Finally, it should be noted that fewer sporozoites were injected by mosquitoes than by needle [1500, assuming an average of 75 sporozoites per mosquito (Medica and Sinnis, 2005) versus 4000 sporozoites by needle], thus accounting for the lower infections observed in the mosquito-bitten mice.

**Fig. 3:**
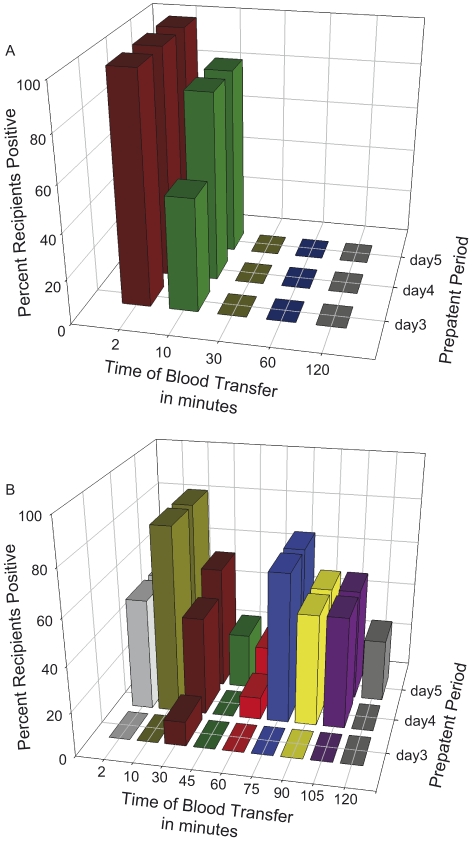
Intradermally injected sporozoites are slowly released from the injection site into the bloodstream. Donor mice were injected with 25 000 *P. yoelii* sporozoites either intravenously (A) or intradermally (B). At the indicated time points after injection, donor mice were exsanguinated by cardiac puncture and one-third of the recovered blood (∼300 μl) was injected intravenously into each recipient mouse, which was then monitored for the presence of blood stage parasites beginning on the third day after inoculation. *x*-axis, the time of blood transfer from the donor; *y*-axis, the proportion of recipient mice that became positive for erythrocytic stage parasites; *z*-axis, the pre-patent period for the mice that became positive. Shown are the combined data from three experiments. There were four to six donors and 10–18 recipients per time point.

These experiments suggested that the majority of infectious sporozoites take hours to exit the injection site. To verify these data and confirm that infectivity does not decline substantially in sporozoites released later rather than earlier after inoculation, we performed blood sub-inoculation experiments. Donor mice were injected with 25 000 sporozoites and at different time points after injection the animals were bled by cardiac puncture. The blood from one donor was divided and inoculated intravenously into each of three naïve recipients. Recipient mice were monitored for blood stage infection in order to determine whether the transferred blood contained infectious sporozoites. In initial experiments, we used intravenously inoculated donors in order to establish that infectious sporozoites could indeed be transferred from donor to recipient by blood transfer and to examine the kinetics with which circulating sporozoites were cleared from the blood. These experiments established that once in the circulation, infective sporozoites are cleared in less than 30 min (Fig. 3A); none of the mice receiving blood collected from donor mice at 30 min or more after sporozoite inoculation, became positive. When sub-inoculation experiments were performed using blood from donor mice that had been injected intradermally with 25 000 sporozoites, the results were strikingly different (Fig. 3B). Irrespective of the time that donor blood was collected, about half of the recipient mice became infected, demonstrating that the pool of inoculated sporozoites in the skin is continuously replenishing the blood circulation with infective sporozoites.

The hepatic stage of *P. yoelii* is completed between 45 and 48 h, ending with the release of merozoites into the blood stream, where they invade red blood cells and multiply every 18 h. When the sporozoite inoculum is large (> 1000 sporozoites) parasites can be observed in the blood starting from day 3 post sporozoite inoculation (Gantt *et al.*, 1998). As shown in Fig. 3A, the majority of recipients given blood from intravenously injected donors had a pre-patent period of 3 days, an expected result given the large numbers of sporozoites that we injected into these donor mice. A 1 day delay in the pre-patent period is considered to indicate a five- to 10-fold decrease in the inoculum and we have previously established that mice inoculated intravenously with 100 sporozoites of the same *P. yoelii* line become patent on day 4 post inoculation (Gantt *et al.*, 1998). Thus, given the observed pre-patent periods in the recipients of blood from intradermally inoculated donors, it is likely that infective sporozoites are transiting from the skin to the blood at a slow steady rate, at least for the first 2 h post inoculation (Fig. 3B).

**Fig. 4:**
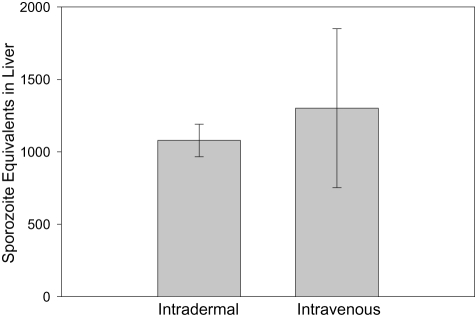
Sporozoite infectivity after intradermal versus intravenous inoculation. Mice were inoculated with 5000 *P. yoelii* sporozoites by intravenous or intradermal injection and 40 h later they were sacrificed and liver parasite burden was quantified by RT-PCR. There were five mice per group and shown are the means ± standard deviations.

In order to determine whether the route of sporozoite inoculation alters the effective inoculum size, 5000 sporozoites were introduced intradermally or intravenously in two separate groups of animals, and the parasite liver load was determined 40 h later. As shown in Fig. 4, the difference in parasite loads was not significant, though variability was higher for animals inoculated intravenously, a finding that was consistently observed (three experiments, four to five mice/group/experiment). The variability observed with intravenously inoculated sporozoites could reflect differences in fitness of the sporozoites reaching the liver. These differences might not be present in sporozoites that have had to go through the dermis.

**Fig. 5:**
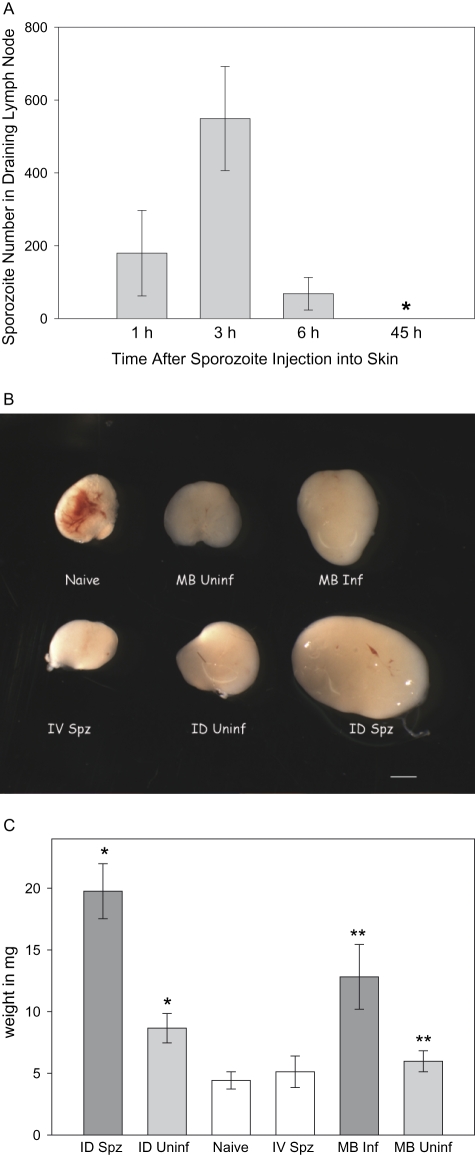
A proportion of intradermally injected sporozoites go to the draining lymph node. A. Five thousand *P. yoelii* sporozoites were injected into one ear of each mouse and at the indicated time points the ipsilateral auricular lymph node was removed and parasite numbers were quantified by PCR. There were five mice per group and shown are the means ± standard deviations. B. Mice were injected in the left ear either intradermally with medium alone (Naive), 5000 *P. yoelii* sporozoites intravenously (IV Spz), salivary gland homogenates intradermally (ID Uninf), or 5000 *P. yoelii* sporozoites intradermally (ID Spz) or fed upon by 10 uninfected mosquitoes (MB Uninf) or 10 infected mosquitoes (MB Inf) and 6 days later the left auricular lymph node was removed, fixed and photographed. Bar = 1 μm. C. Lymph nodes from the mice in B were weighed. There were five mice per group and shown are the means ± standard deviations. Student's *t*-test for unpaired data showed that the weights of the lymph nodes of mice injected intradermally with sporozoites was significantly different from those of mice injected intradermally with uninfected salivary glands (*P < 0.001) and the weights of lymph nodes of mice bitten by infected mosquitoes was significantly different from those bitten by uninfected mosquitoes (**P < 0.01).

Next we wished to determine whether *P. yoelii* sporozoites trafficked to the draining lymph node after their injection into the skin. We therefore injected *P. yoelii* sporozoites intradermally into mouse ears and performed PCR quantification of total genomic DNA purified from the dissected ipsilateral auricular lymph node that drains the ear injection site. As shown in Fig. 5A, approximately 20% of the *P. yoelii* sporozoites reach the lymphatics. The kinetics of appearance of sporozoites in these lymph nodes parallels that of the migration out of the inoculation site, in that the largest numbers of sporozoites appear in the lymph node 3 h after inoculation. By 6 h post injection, few sporozoites are detectable in the lymph nodes. As lymph nodes are an important site of immunological priming, we wished to ascertain whether transit of sporozoites altered the lymph nodes. To this end, we measured the size of the lymph nodes draining the ear in which sporozoites were inoculated 6 days previously. Significant enlargement of these lymph nodes was specifically associated with the site of intradermal inoculation as the lymph nodes draining the uninjected ear were not enlarged (data not shown). In addition, intravenous inoculation of equal numbers of sporozoites did not result in lymph node enlargement (Fig. 5B and C). The observed enlargement was not accounted for by the salivary gland debris present in the inoculum, because the lymph nodes of mice injected with material obtained from an equivalent number of uninfected mosquitoes were found to be significantly less enlarged (*P* < 0.001; Fig. 5B and C). When sporozoites were inoculated by infected mosquitoes, the draining lymph node was also enlarged, however, not to the same degree observed with intradermal injection. These data suggest that the extent of enlargement is correlated to the number of sporozoites transiting through the lymph nodes because a median of 750 sporozoites would be expected to be inoculated by 10 infected mosquitoes, as compared with the 5000 injected intradermally. The anterior cervical lymph nodes, the nodes distal to those draining the ear, were not enlarged in the mice receiving 5000 sporozoites intradermally (data not shown). This suggests that the sporozoites draining to the lymphatics are destroyed, though this remains to be formally demonstrated.

## Discussion

The data presented in this study uncover two hitherto unsuspected facets of the biology of the *Plasmodium* sporozoite. The infective sporozoites inoculated by the mosquito are released from the skin into the blood circulation in a trickle extending for hours after the mosquito bite. Thus, sporozoites retain infectivity for prolonged periods following their transition to the mammalian host. It is likely that a factor(s) in the skin preserves the infectivity of the inoculated sporozoites for a number of hours. This is in contrast to the rapid loss of infectivity observed in sporozoites placed *in vitro* at 37°C in physiological media. Our data raise the possibility that the other infective form in the mammalian host, the merozoite, might also retain infectivity *in vivo* for longer periods than has been assumed from *in vitro* observations.

A recent study showed that saliva introduced into the skin during mosquito feeding can trigger degranulation of cutaneous mast cells and dermal infiltration by neutrophils (Demeure *et al*., 2005). As the majority of sporozoites spend 1–2 h at the injection site, they may encounter these activated cells. The effect of these mosquito-induced innate immune responses on sporozoite infectivity remain to be elucidated. Specifically, it will be of interest to know the precise timing of mast cell degranulation and neutrophil infiltration in order to determine the role, if any, that innate immunity plays in the very early stages of malaria infection. There is a suggestion in our data that sporozoites remaining in the skin after 3 h are destroyed as we consistently see robust sporozoite signals in the skin beyond 3 h (Fig. 1A) but fail to see a statistically significant increase in liver infectivity when the injection site is removed 3 h after sporozoite inoculation (data not shown). Undoubtedly this will be a fruitful area of future investigation.

The data we present here with *P. yoelii* are largely complementary to the recently published study in which early events in *P. berghei* sporozoite infection were studied by intravital microscopy (Amino *et al*., 2006). In that study, however, sporozoites were observed to lose motility by 1 h after their inoculation, suggesting that they were no longer infectious after this time. The difference between our studies could reflect differences between the biology of the two *Plasmodium* species used or differences in the skin environment of the mouse strains used. Another possible explanation is that prolonged microscopic observation of the inoculation site led to an increase of temperature which adversely affected sporozoite motility.

There are two studies in humans relevant to our observations in the mouse model. First, in a seminal study where the length of the pre-erythrocytic cycle was established (Fairley, 1947), the presence of infectious sporozoites in the blood was assessed by transfusion of blood from donors exposed to the bites of *Plasmodium falciparum*- or *Plasmodium vivax*-infected mosquitoes into naïve recipients. In one set of experiments, transfusions were performed at times ranging from ‘during biting’ to 2 h after biting ceased. Seven of a total of 16 recipients became positive for blood-stage parasites and two of the seven were transfused with blood at later time points, i.e. 30 min and 60 min after cessation of mosquito biting. In the other experiments, transfusions were carried out with blood collected on days 1–8 after mosquito biting and none of these recipients became infected. Although these observations do not offer conclusive demonstration, they are consistent with the occurrence of a trickle of sporozoites from the skin into the circulation. However, there are limitations inherent in using human volunteers that limit the types of data one can gather. Fairley was restricted in the number of human volunteers he could use, the number of infective bites to which human subjects were exposed, and the volume of blood he could withdraw from the donor and transfuse into the recipient. Using the rodent system we were able to increase the initial sporozoite inoculum by an order of magnitude, to inject a much larger percentage of the donor's blood volume into recipients (75% of the donor's blood volume into three recipients versus 4–10% of the donor's blood volume into one recipient) and to use larger numbers of recipients (10–18 recipients per time point versus one to four recipients per time point), all of which likely contributed to our ability to quantitatively observe infectious sporozoites as they entered the blood circulation. The other study of relevance to our work is the work of Boyd and Kitchen (1939) who observed intact *P. vivax* sporozoites in a lymph node 24 h after mosquito bite, a clear indication that sporozoites can survive outside the blood stream for long periods of time. Taken together these observations suggest that sporozoites might behave in a similar manner in humans and rodents.

The slow release of sporozoites from the skin into the circulation and the finding of a proportion of the inoculum in the lymph node are of potential significance to current efforts to develop and optimize vaccines targeting the pre-erythrocytic stages of *Plasmodium* (Luke and Hoffman, 2003; Hill, 2006). Prolonged residence of sporozoites in the skin adds to the time during which this extracellular parasite form would be exposed to immune attack, and comforts the notion that induction of neutralizing antibodies to sporozoites should remain a focus of the malaria vaccine community. Exposure of antigen presenting cells, present in the lymph nodes, to significant amounts of sporozoite antigens raises the possibility that sporozoites introduced into the mammalian host by natural infection may induce a qualitatively different immune response than that induced by intravenous inoculation.

## Experimental procedures

### Parasites

Three- to five-day-old *Anopheles stephensi* mosquitoes were fed on anaesthetized *P. yoelii* (clone 17X)-infected Swiss Webster mice with abundant gametocyte-stage parasites. Salivary gland sporozoites were harvested on days 14–15 post infective blood meal. The mosquitoes were rinsed in 70% ethanol and washed in Dulbecco's Modified Eagle Medium (DMEM) before salivary gland dissection. The glands were gently homogenized, spun at 80 *g* for 3 min, to remove mosquito debris, the sporozoite-containing supernatant was removed and sporozoites counted in a haemocytometer.

### Quantification of parasites in the skin and lymph node

Four- to six-week-old Swiss Webster mice were anaesthetized and maintained at 37°C on a slide warmer. A total of 5000 sporozoites (in 5 μl DMEM) were inoculated by intradermal injection into the pinna of the ear with a Flexifill microsyringe (World Precision Instruments, Sarasota, FL). The injected ears and ipsilateral auricular lymph nodes were removed at the indicated time points, weighed, cut into small pieces, and digested overnight at 50°C with proteinase K. Genomic DNA was extracted using the DNeasy tissue kit (QIAGEN, Valencia, CA) and parasite genomic DNA was quantified using nested PCR followed by real-time PCR. For nested PCR, we used 100 ng of total genomic DNA and primers specific for the *P. yoelii* 18S rRNA; forward primer, 5′-ctaattagcggcgagtacgctatatcc and reverse primer, 5′-ctaaggacatcacagacctgttgtttgc. These sequences are found 67 bp upstream and 34 bp downstream, respectively, of the primers used in the subsequent real-time PCR. The cycling profile was 95°C for 3 min followed by 15 cycles of: 95°C, 30 s; 60°C, 30 s; 72°C, 30 s; and a final extension of 72°C for 5 min. Quantitative PCR analysis was then performed in a Rotor-Gene 3000 (Corbett Life Science, Sydney, Australia) using 5 μl of the nested PCR, 5 ρM each of forward (5′-ggggattggttttgacgtttttgcg) and reverse (5′-aagcattaaataaagcgaatacatccttat) primers (Bruna-Romero *et al*., 2001) and the QuantiTect SYBR green PCR kit (Qiagen, Valencia, CA). Parasite numbers were determined using a standard curve made from mouse ears or lymph nodes spiked with 0, 200, 1000 and 5000 sporozoites. Genomic DNA was isolated and processed from these samples as described for the experimental tissue samples.

### Quantification of parasites in the liver

Swiss Webster mice were anaesthetized and maintained at 37°C on a slide warmer. Four thousand sporozoites were inoculated by intradermal injection with the Flexifill microsyringe into the pinna of one ear. At the indicated time points, mice were anaesthetized again and the injected ear was removed using a surgical scissors under aseptic conditions. Control mice had the uninjected ear removed 3 h post sporozoite inoculation. Forty hours after sporozoite inoculation, the livers of all mice were harvested, total RNA was isolated using Tri-reagent (Molecular Research Center, Cincinnati, OH) and the parasite burden in the liver was quantified using reverse transcription (RT) followed by real-time PCR as outlined in Bruna-Romero *et al*. (2001). RT was performed using 1 μg of total RNA and random hexamers and real-time PCRs were performed using primers that recognize *P. yoelii*-specific sequences within the 18S rRNA (Bruna-Romero *et al*., 2001) and the QuantiTect SYBR Green PCR Kit (QIAGEN, Valencia, CA). Liver infection was quantified by comparison with a standard curve made from livers of mice that had been injected intravenously with 10, 100, 1000 or 10 000 sporozoites. For the experiments in which sporozoites were injected by mosquito bite, 10 mosquitoes were allowed to probe for 3 min on each ear. Both ears were removed at the indicated time points and control mice did not have their ears removed. In experiments where sporozoites were injected by mosquito bite into the back, the hair was removed from the back 1 day prior to the experiment and the skin was pinched up during feeding so that sporozoites would only be deposited in the dermis and not in the muscle layer beneath the dermis. Twenty mosquitoes were allowed to probe on an area that was 1 cm in diameter which was then removed at the indicated time points. In control mice a 1 cm area adjacent to the probed-upon area was removed. Removal of the injection site or control area was performed in a sterile dissection hood using aseptic technique on anaesthetized mice and the wound was closed with 3.0 silk sutures.

### Sub-inoculation experiments

Six-week-old Balb/C mice were anaesthetized, maintained on a slide warmer at 37°C and injected either intravenously or intradermally with 2.5 × 10^4^*P. yoelii* sporozoites. Shortly before the indicated time points the mouse was anaesthetized again and then bled by cardiac puncture using heparin (100 units ml^−1^) as an anticoagulant. Between 0.6 and 0.9 ml of blood was obtained from each mouse. Three hundred microlitres of collected blood was inoculated intravenously into each recipient Balb/C mouse. Recipients were monitored for the presence of erythrocytic stage parasites by Giemsa-stained blood smears until day 20 after blood inoculation.

### Lymph nodes

Mice were inoculated intradermally in the ear with either 5000 *P. yoelii* sporozoites, an equivalent amount of salivary gland material from uninfected mosquitoes, the bites of 10 *P. yoelii*-infected mosquitoes, the bites of 10 uninfected mosquitoes or medium alone. Another group was injected intravenously with 5000 *P. yoelii* sporozoites. Six days later the ipsilateral auricular lymph node was removed from each mouse, fixed in 4% paraformaldehyde at 4°C, weighed, measured and photographed with a Leica M3Z dissecting microscope with an attached Optronics CCD camera, using Magnafire 2.0 image software.
